# Pro-inflammatory cytokine alterations in unaffected first-degree relatives of schizophrenia patients

**DOI:** 10.1192/j.eurpsy.2021.403

**Published:** 2021-08-13

**Authors:** A. Kurtulmus

**Affiliations:** 1 Psychiatry, Istanbul Medeniyet University Goztepe Research and Training Hospital, Istanbul, Turkey; 2 Psychiatry, Bezmialem Vakif University, Istanbul, Turkey

**Keywords:** endophenotype, cytokines, schizophrénia

## Abstract

**Introduction:**

A growing body of evidence in both chronic and first-episode schizophrenia report increased expression of pro-inflammatory substances in the blood and cerebrospinal fluid of patients. However, there is not much data in the literature on immune alterations in unaffected first-degree relatives (FDRs) of the patients.

**Objectives:**

We aimed to evaluate inflammatory aberrancies in patients with schizophrenia, their unaffected first-degree relatives (FDRs) and healthy controls.

**Methods:**

50 chronic, stable schizophrenia patients, 42 FDRs and 40 healthy subjects with no family history (HCSs) were recruited to the study. IL-1β, IL-6, TNF-a and CRP levels were measured. Complete blood counts, fasting glucose and lipid levels were analyzed and neutrofil-lymohocyte ratio (NLR) were calculated.

**Results:**

There was a significant group difference in all cytokine levels after controlling for age, gender, smoking status, comorbid medical diseases, BMI and blood glucose and tyrigliseride levels (p<.001). FDRs showed significantly higher serum levels of cytokines than HCs, in the same way as the corresponding schizophrenia patients but a lower level. Pairwaise comparisions revealed that the differences were significant between each group after controlling for confounders (p<.001 for all comparisons). However, NLR and CRP levels were not different between groups.

**Conclusions:**

Our results support the role of inflammatory aberrancies in the pathophysiology of schizophrenia. The finding of abnormal cytokine levels both in schizophrenic patients and FDRs indicates that such immunological alterations are not exclusive to the patients and can be possible endophenotypes for the disorder.

**Disclosure:**

No significant relationships.

